# Ultrastructural changes in methicillin-resistant *Staphylococcus aureus* induced by positively charged silver nanoparticles

**DOI:** 10.3762/bjnano.6.246

**Published:** 2015-12-15

**Authors:** Dulce G Romero-Urbina, Humberto H Lara, J Jesús Velázquez-Salazar, M Josefina Arellano-Jiménez, Eduardo Larios, Anand Srinivasan, Jose L Lopez-Ribot, Miguel José Yacamán

**Affiliations:** 1 Department of Physics and Astronomy, The University of Texas at San Antonio, One UTSA Circle, San Antonio, Texas 78249, USA; 2 Departamento de Ingeniería Química y Metalurgia, Universidad de Sonora, Rosales y Luis Encinas S/N, Hermosillo, Sonora C.P. 83000, México; 3 Department of Biology and South Texas Center for Emerging Infectious Diseases, The University of Texas at San Antonio, San Antonio, Texas 78249, USA

**Keywords:** electron microscopy, methicillin-resistant *Staphylococcus aureus* (MRSA), positively charged nanoparticles, silver nanoparticles, *Staphylococcus aureus*, wall teichoic acids

## Abstract

Silver nanoparticles offer a possible means of fighting antibacterial resistance. Most of their antibacterial properties are attributed to their silver ions. In the present work, we study the actions of positively charged silver nanoparticles against both methicillin-sensitive *Staphylococcus aureus* and methicillin-resistant *Staphylococcus aureus*. We use aberration-corrected transmission electron microscopy to examine the bactericidal effects of silver nanoparticles and the ultrastructural changes in bacteria that are induced by silver nanoparticles. The study revealed that our 1 nm average size silver nanoparticles induced thinning and permeabilization of the cell wall, destabilization of the peptidoglycan layer, and subsequent leakage of intracellular content, causing bacterial cell lysis. We hypothesize that positively charged silver nanoparticles bind to the negatively charged polyanionic backbones of teichoic acids and the related cell wall glycopolymers of bacteria as a first target, consequently stressing the structure and permeability of the cell wall. This hypothesis provides a major mechanism to explain the antibacterial effects of silver nanoparticles on *Staphylococcus aureus.* Future research should focus on defining the related molecular mechanisms and their importance to the antimicrobial activity of silver nanoparticles.

## Introduction

Bacterial infections are a major reason of morbidity and mortality globally [1], and most infections can be attributed to species of the genus *Staphylococcus* [2]. *Staphylococcus aureus* (*S. aureus*) is well known for its ability to acquire genetic resistance against almost all antibiotics [3]. As penicillin and other β-lactams were previously very efficient antibiotics in treating staphylococcal infections, the prevalent resistance of methicillin-resistant *Staphylococcus aureus* (MRSA) has made therapy continuously more complex [4]. *S. aureus* has also become resistant to antibiotics of last resort, including vancomycin [5], daptomycin [6], and linezolid [7]. β-Lactam antibiotics target the synthesis of peptidoglycan (PG), a cell wall polymer that renders structural strength and counteracts the osmotic pressure of the cytoplasm, known as turgor pressure. MRSA is resistant to all ß-lactam antibiotics due to its production of an extra penicillin-binding protein (PBP2a) [8]. With scarce management options for MRSA, there is a pressing necessity for the development of novel bactericides [9].

*S. aureus* is capable of causing chronic bone and joint infections [10]. It has been acknowledged that the emergence of new MRSA strains amongst clinical isolates conveys the single most critical obstacle to the pharmacological treatment of *S. aureus* infection [11]. *S. aureus* resistance is frequently associated with hospital-acquired MRSA (HA-MRSA) or community-associated MRSA (CA-MRSA) infections having substantially higher rates of mortality [12]. Therefore, antibiotic resistance has developed into a serious universal health risk [13]. Antibiotic resistant infections are becoming progressively more prevalent during hospitalizations in the United States, with a trend that increased by 359% between 1997 and 2006 [12]. The ongoing prevalence of MRSA and the appearance of other resistant strains create urgency for the development of novel treatments [14]. However, in the past years, antibiotic developments have brought only partial success [15]. Select bactericidal agents are preferred for severe infections, for instance endocarditis or meningitis [16].

The bacterial cell wall is important for maintaining structural support and for providing shelter against osmotic cell lysis. Gram-positive peptidoglycan (PG) embodies various layers and measures from 30 to 100 nm. Layers of PG are built by anionic glycopolymers, known as wall teichoic acids (WTAs) [17]. These WTAs are essential in maintaining bacterial architecture, replication, and other main cell functions [18]. WTAs play an important role in antibiotic resistance in MRSA, and they increase bacterial vulnerability to cationic antimicrobials, peptides, ions and metals. WTAs retain surface proteins by either covalent or noncovalent attachment [19]. Electron cryomicroscopy studies on *S. aureus* indicate that WTAs extend over the PG layer. Disregarding their differences, all WTAs maintain anionic backbones, which always carry negative charge [20].

A serious risk factor for *S. aureus* infections is nasal carriage. Although the exact mechanisms that account for bacterial settlement in the nasal epithelium are still not well established, one essential element is the WTA of *S. aureus*, which governs direct interactions with nasal tissue surfaces in a charge-dependent manner [21]. It has been postulated that WTAs can attach to metal cations by spreading outside of the layers of PG [22] and consequently that cells lacking WTAs show a decreased proton-binding capability [20].

The bactericidal effect of silver is widely known. Around the 1800s silver nitrate was commonly applied topically to treat burns and ulcerations or infected wounds, although its use declined following the introduction of antibiotics. Fox revived its use in the form of silver sulfadiazine, which is applied topically in burn therapy [23]. An encouraging path of research in nanobiotechnology involves silver nanoparticles (AgNPs) [24]. AgNPs are potent bactericidal agents with broad-spectrum activity [25]. In fact, the number of papers on this topic has exploded during the last decade [26–33]. AgNPs also have antifungal [34] and antiviral [35–37] activities. Due to their small-scale diameters and enhanced surface area to volume ratios, metallic nanoparticles have large contact areas available to interreact with pathogens [24]. AgNPs can disturb the physiology of bacterial cell membranes by affecting their permeability [38]. After penetrating the cell membrane, AgNPs can also alter sulfur-containing amino acids and phosphorus (a main constituent of DNA), inhibiting replication via attaching to the bacterial ribosome [39–40]. The proteomic signatures of AgNP-treated *E. coli* demonstrated an accumulation of envelope protein precursors, demonstrating that AgNPs target bacterial cell membranes by depleting the proton motive force.

AgNP surfaces adsorb Ag^+^; therefore, colloids consist of three species of silver: Ag^+^, metal Ag^0^ (its mixture), and surface-adsorbed Ag^+^ [41]. Recently, the presence of three particle species in a citrate-stabilized nanosilver colloidal solution was reported: neutral AgNPs (Ag^0^), silver ions (Ag^+^) and Ag^+^ adsorbed on Ag^0^ (Ag^0^/Ag^+^). Battharai et al. was able to show the presence of Ag^0^ by performing a TEM investigation. Additionally, the existence of Ag^+^ adsorbed on Ag^0^ was discovered, and it was established that as Ag ions coalesce, as they formed AgNPs carrying an extra positive charge, which was that of Ag^+^ [42]. Similarly, this occurs in AgNPs that contain mixtures of Ag^0^ and Ag^+^; as Ag^+^ coalesces into larger Ag particles, these new particles become positively charged. This type of AgNP species is referred to as the third species, and it is central to the study of the bactericidal and ultrastructural effects of AgNPs against methicillin-sensitive *Staphylococcus aureus* (MSSA) and MRSA.

By acquiring zeta potential measurements before and after the filtration of Ag colloids, Bhattarai et al. showed a shift in potential from −46.0 mV to −20.9 mV. The strong ionic presence in the solution after filtration was responsible for the peak shift. Furthermore, the presence of three species in an AgNP solution is important, as we propose a new mechanism for how each of these three species interacts with the bacterial cell [42].

The complex action mechanism of silver metals decreases the probability of bacteria developing a resistance against it, even though several resistance mechanisms to metals have been described [43], the most common of which is metal ion efflux. This was demonstrated by research on the development rate of spontaneous mutations in response to silver compounds in *S. aureus*. Silver-resistant mutations were not identified when silver sulfadiazine was applied as a bactericidal agent [44–45]. The establishment of microbial resistance to silver via bacterial plasmids has been studied by geneticists at a molecular level [46]. The continuous leaching of silver ions by AgNPs [42] creates a favorable antimicrobial environment.

MSSA and MRSA were treated with a solution of AgNPs to study the ultrastructural changes and bactericidal and lytic effects that were induced by AgNPs, which were investigated using various electron microscopy techniques, such as high resolution transmission electron microscopy (HRTEM), high angle annular dark field scanning transmission electron microscopy (HAADF-STEM), scanning electron microscopy (SEM), and energy dispersive X-ray spectroscopy (EDS), along with calculating the MIC_50_ inhibitions of AgNPs on MRSA and MSSA as well as zeta potential measurements to investigate the charged-particle nature of silver nanoparticles. The study revealed that our 1 nm average sized AgNPs induced pore formation, cell wall thinning, cell content leakage and cell lysis with growth inhibition in a dose-dependent manner after the particles attached beyond the PG layer. We hypothesize that positively charged silver nanoparticles bind to the negatively charged polyanionic backbones of teichoic acids and cell wall glycopolymers (CWGs) as first targets, leading to structural strain in and permeability of the bacterial cell wall. This finding provides a major mechanism to explain the antibacterial properties of silver nanoparticles on *Staphylococcus aureus.*

## Results and Discussion

### Characterization of AgNPs

**Figure 1:**
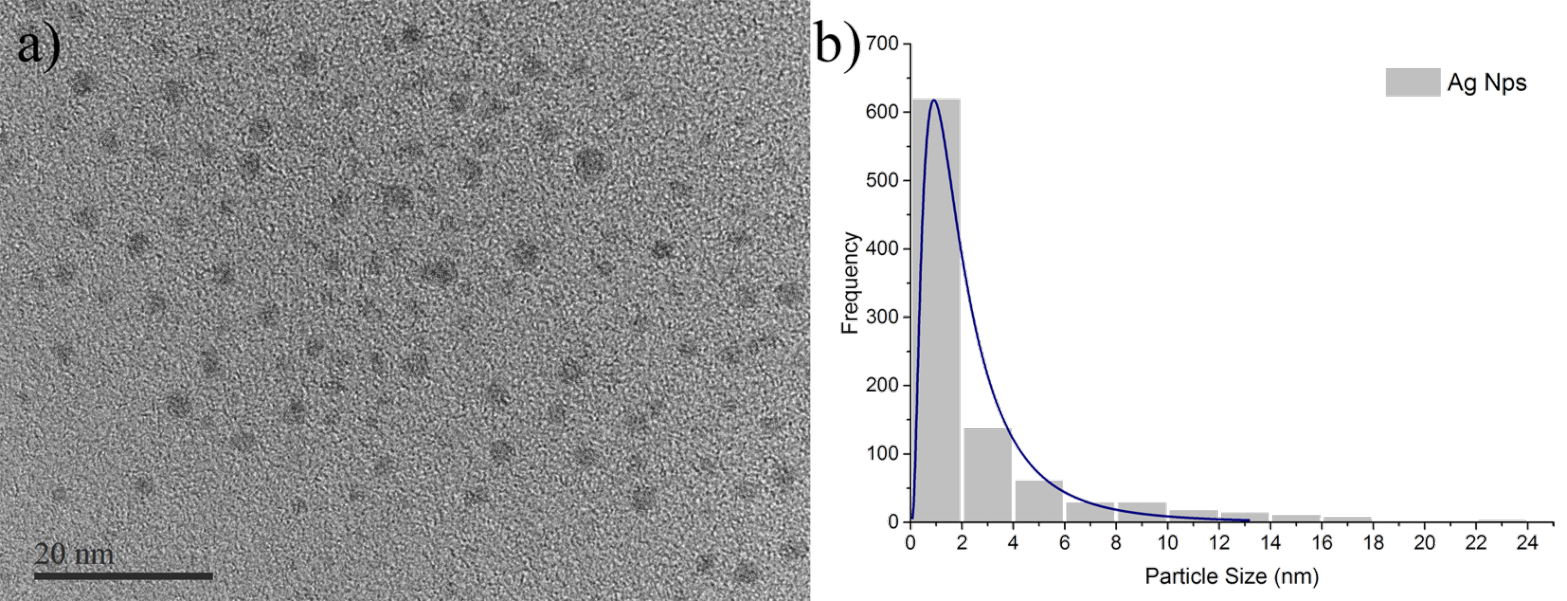
(a) TEM micrograph of AgNPs. (b) Log–normal size distribution graph shows the average AgNP size of approximately 1 nm.

TEM images of silver nanoparticles (Figure 1a). The measured distribution was log–normal distributed with a size distribution range of 0.5 to 24 nm and average size of 1 nm (Figure 1b).

### Zeta potential measurements

**Figure 2:**
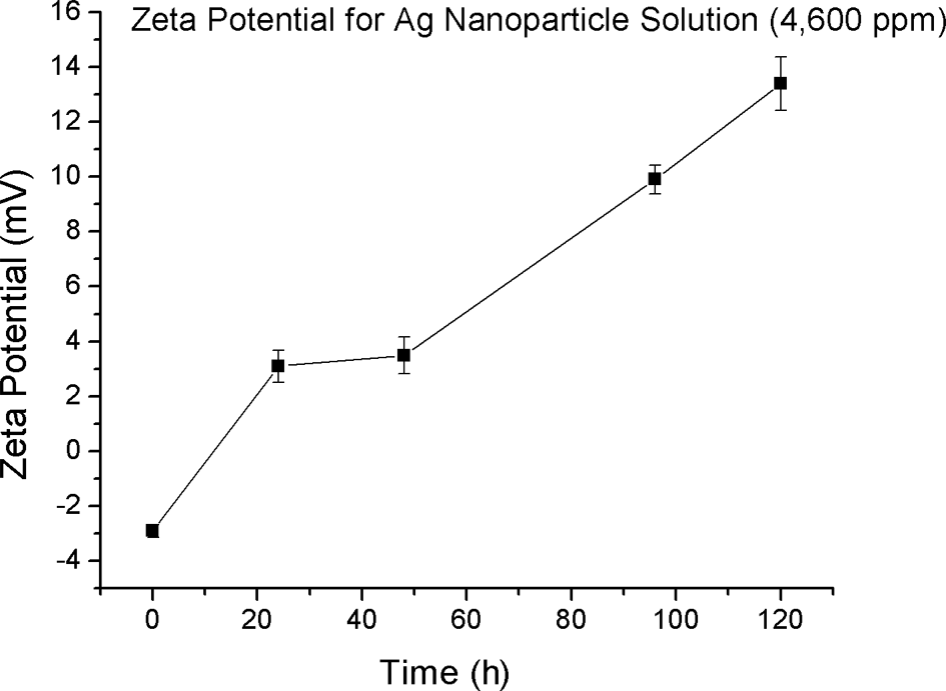
Zeta potential of AgNP solution (4,600 ppm) over a time of 120 h.

The zeta potential value increased with time from −2.9 mV to +13.4 mV over a 120 h time period (Figure 2). This shift to positive zeta potential indicates the adsorption of cations from the environment onto the particles [47]. These data suggest that silver nanoparticles become positively charged, leading to their aggregation and enlargement over time.

### Electron microscopy of untreated MRSA cells

**Figure 3:**
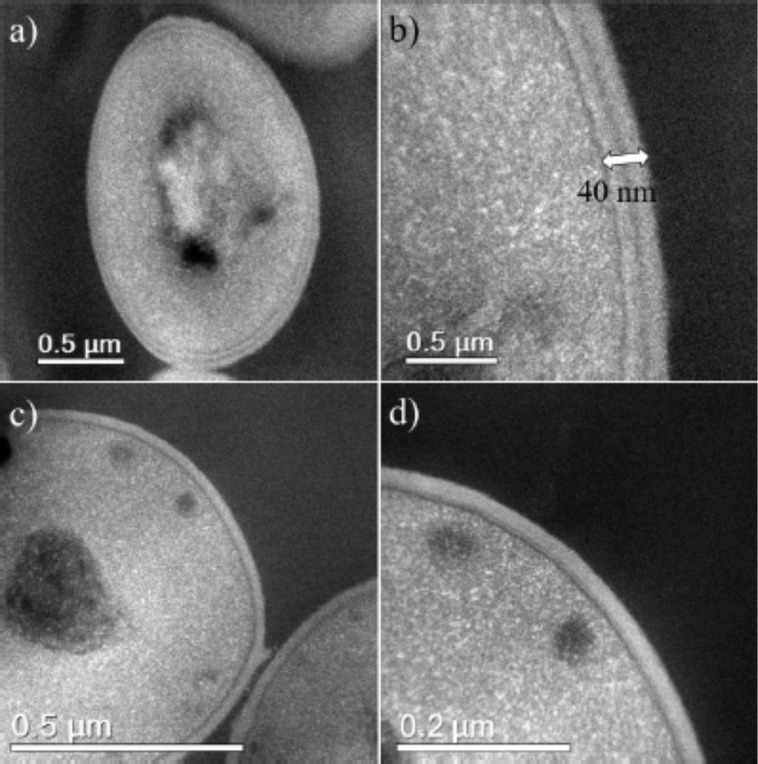
(a) HAADF-STEM image of an untreated MRSA cell. (b) High magnification image of a MRSA cell wall containing peptidoglycan layers with a total thickness of 40 nm. (c) An undamaged MSSA cell wall. (d) A high magnification image of a MSSA cell wall showing the cell envelope layers.

Untreated MSSA and MRSA cells showed large areas where osmium did not penetrate, as seen in Figure 3. The bacterial cells are generally intact. Furthermore, the cell walls of the untreated MRSA and MSSA cells seem rigid, and their PG layers are thick, measuring approximately 40 nm compared to approximately 32 nm in the case of the treated bacterial cells (see below in Figure 7b).

### Electron microscopy of treated MSSA and MRSA

**Figure 4:**
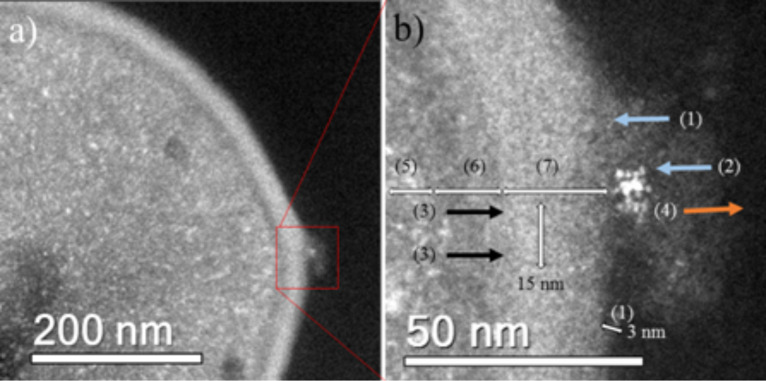
MSSA cell STEM image. (a) MSSA cell showing AgNP attachment on the cell wall. (b) High magnification STEM images of MSSA cell (1) wall teichoic acids (≈3 nm), (2) interaction of AgNPs with CWGs, (3) destabilization, (4) leakage of cytoplasmic material leading to bacterial cell lysis, (5) cytoplasm, (6) cytoplasmic membrane and (7) cell wall. The AgNP concentration was 11.5 ppm.

**Figure 5:**
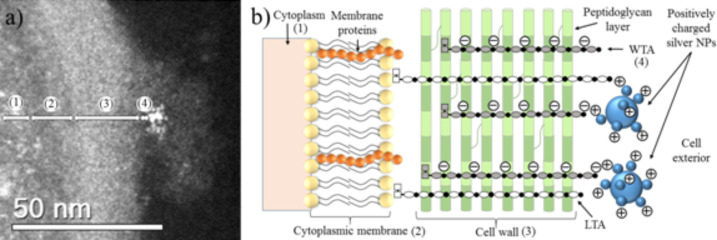
STEM micrograph of cell envelope of MSSA treated with AgNPs. (1) Cytoplasm, (2) cytoplasmic membrane, (3) cell wall and (4) wall teichoic acids. (b) Schematic diagram of our hypothesis on the interaction between AgNPs and the teichoic acids of the cell wall. (WTA = wall teichoic acids; LTA = lipoteichoic acids). The AgNP concentration is 11.5 ppm.

**Figure 6:**
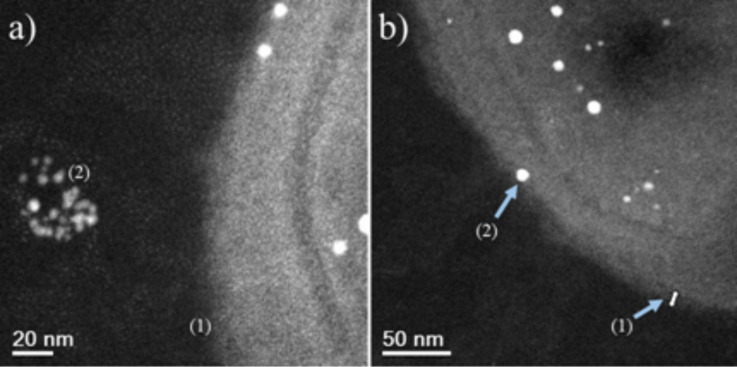
MSSA STEM micrographs. (a,b) (1) WTAs and CWGs (≈3 nm). (2) Interactions between AgNPs and a cell wall. The Ag nanoparticle concentration is 23 ppm.

**Figure 7:**
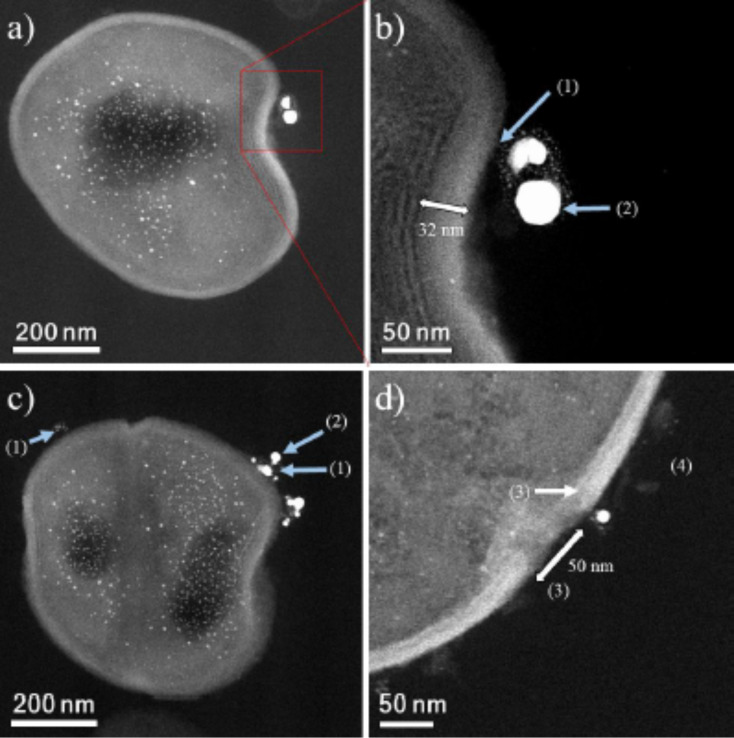
HAADF-STEM micrographs of MRSA cells. (a) MRSA cells surrounded by AgNPs, with AgNPs smaller than 10 nm also being found inside of the cells. (b,c) (1) CWGs. (2) AgNPs interacting with WTAs and CWGs. MRSA cell micrograph shows a cell wall size of 32 nm. The Ag nanoparticle concentration is 23 ppm. (d) (3) Membrane destabilization (≈50 nm).

**Figure 8:**
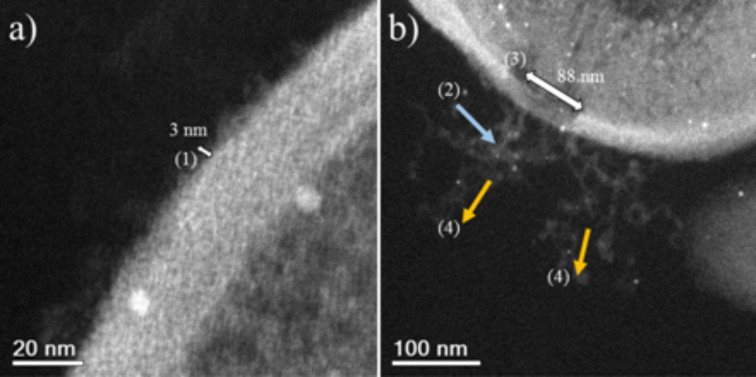
MRSA STEM images. (a) (1) WTAs and CWGs (≈3 nm). (b) (2) AgNPs interacting with CWGs. (3) Membrane destabilization (≈88 nm). (4) Cytoplasmic leakage. The Ag nanoparticle concentration is 23 ppm.

**Figure 9:**
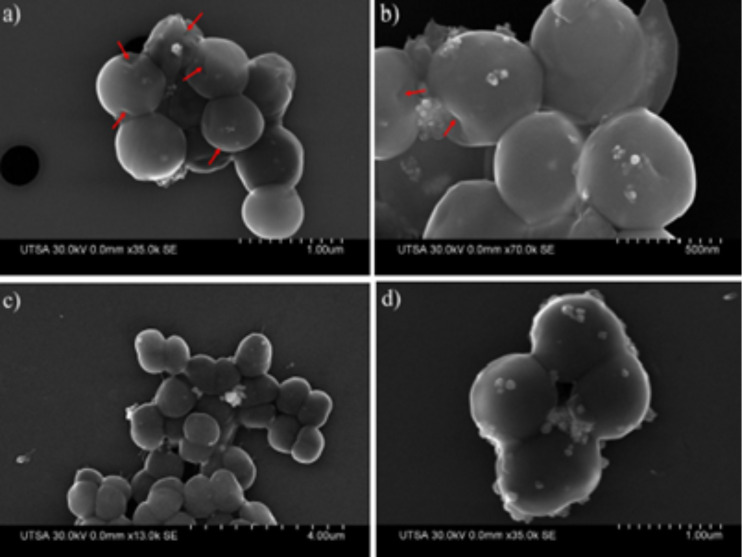
SEM images show cell size and morphology along with AgNPs interacting to the cell surface for (a) and (b) MRSA (arrows point to hole formation) and (c) and (d) MSSA cells. AgNPs concentration 23 ppm.

HAADF-STEM images of treated MRSA and MSSA cells demonstrate the affinity between silver and osmium that can generate electron-dense particles around the bacterial cell wall, as seen in Figure 4a; specific cases of AgNPs binding to cell walls are shown in Figure 4, Figure 5a and Figure 6. Additionally, Figure 4, Figure 5a and Figure 6 show interactions between AgNPs and a cell wall leading to cytoplasmic leakage, which is depicted in the schematic diagram shown in Figure 5b. In Figure 7, the manner in which the *S. aureus* cell wall becomes deformed and damaged is shown. Disruption of bacterial membranes induces pore and hole formation (Figure 9a,b) and also generates deformation of cell shape (Figure 7 and Figure 9a), damage of the PG layer, porosity of the cell membrane and consequent discharge of cytoplasmic (Figure 4b and Figure 8b) material, which eventually precipitates bacterial cell lysis. AgNPs smaller than 10 nm can also be observed inside of cells, whereas larger AgNPs remain outside of cells due to aggregation (see below in Figure 11). Ultramorphological changes occur after AgNP treatment induces damage to the MRSA cell wall, resulting in deformation and eventual cell bursting when AgNPs cluster around the cell wall (Figure 7a,b).

SEM images were also obtained to compare MRSA and MSSA cells treated with AgNPs. In these images, we can see groups of whole cells and their sizes and morphologies, as well as AgNPs attached to cell walls (Figure 9). We observed clear alterations of the cell surfaces (e.g., a change from a smooth to a wrinkled appearance, loss of turgidity, the development of holes (arrows), and outer membrane burst) throughout cell lysis.

**Figure 10:**
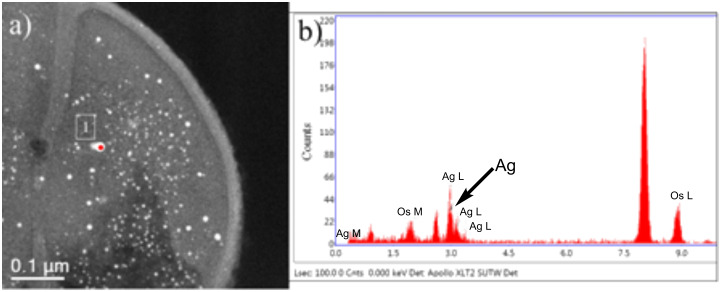
(a) AgNPs inside of a MRSA cell. EDS analysis was performed on one particle, label 1. (b) The EDS spectrum for particle 1 shows silver (Ag) peaks. The AgNP concentration is 23 ppm.

Select regions on the interiors and exteriors of the MRSA bacterial cells confirm the presence of AgNPs, as seen in the EDS spectra (Figure 10). It was noted that AgNPs that interacted with the exteriors of the MRSA cells were larger (Figure 7a,c), whereas those in the cell interiors were smaller (less than 10 nm), as seen in Figure 10a. The EDS spectra confirmed the presence of silver.

### Silver nanoparticles size distribution inside and outside of *S. aureus* cells after treatment

**Figure 11:**
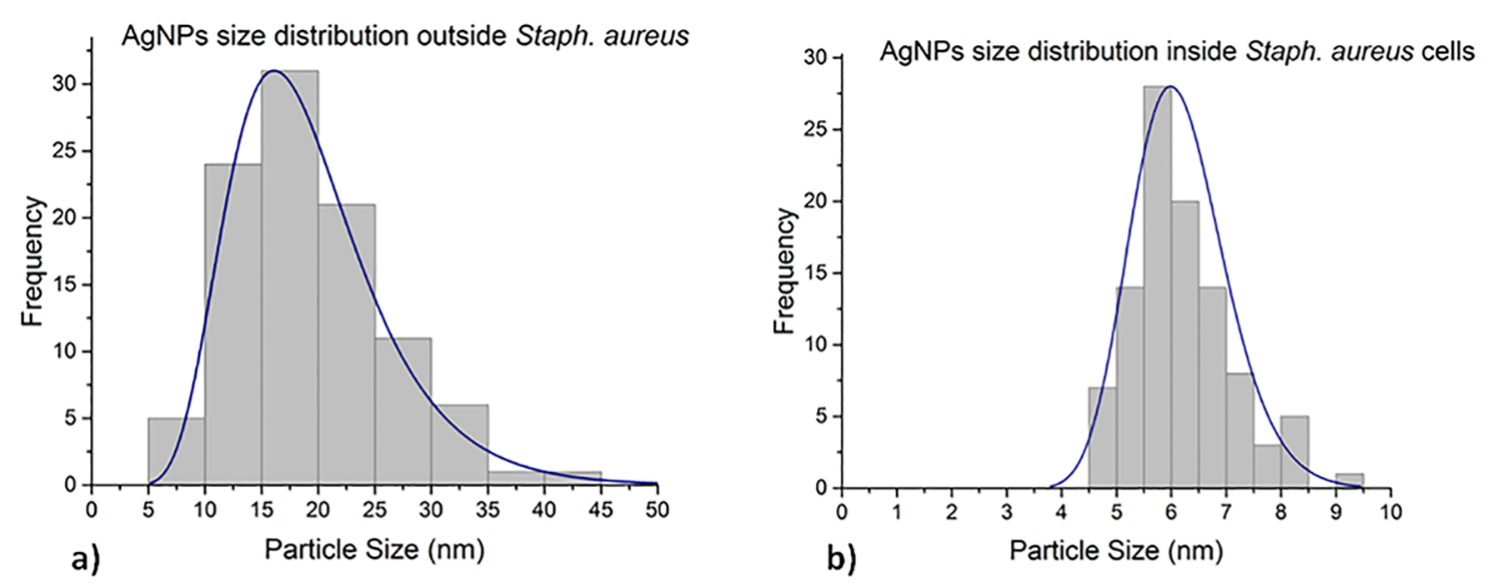
AgNP size distributions for particles (a) inside and (b) outside of *S. aureus* cells after treatment. The histograms were fitted to a log–normal size distribution.

The AgNPs were log–normal distributed in the interiors and on the exteriors of *S. aureus* cells 24 h after treatment. This is shown in Figure 11. The size range of interior AgNPs is 5 to 9 nm, whereas those found on the exteriors the cells after treatment have a size range of 9.5 to 33.0 nm. The mean size values were 5.9 and 18.6 nm, respectively.

### Minimal inhibitory concentration (MIC)

The AgNP MIC against both MSSA (UAMS1) and MRSA (TCH1516) was 12.5 ppm. STEM and EDS were used to examine ultrastructural changes in bacteria induced by AgNPs. Both MSSA and MRSA bacteria were treated with 1 nm average size positively charged AgNPs (Figure 1). After treatment, the bacterial cells appeared wrinkled compared to the untreated controls, which were intact with thick PG layers and firm, rounded cell walls (Figure 3). The ultrastructural alterations of the bacterial cells after treatment implies that AgNPs increase osmosis through permeabilization of the PG cell wall (Figure 9) before they ultimately induce an extreme efflux of cellular contents [48]. The negative impact on bacterial osmoregulation could be associated with the permeabilizing activities of the AgNPs and silver ions on the membrane.

As shown, AgNP treatment can lead to a reduction of the PG layer (which provides strength to the cell wall), consequently generating destabilization and permeabilization of the bacterial cell membrane and causing osmotic rupture and lysis (Figure 4b, Figure 7d, Figure 8b and Figure 9a).

WTAs and related CWGs have important functions in the cell architecture, replication, and other main characteristics of Gram-positive bacteria [20]. Therefore, membrane integrity is vital, and its disruption induces metabolic impairment and cell death. Treating bacteria with AgNPs resulted in an imbalance of the PG layer, as the cells lost the ability to protect themselves against variations in turgor pressure [49].

It is generally accepted that cationic compounds (peptides, antibiotics and metal ions) have the tendency to selectively interreact with and bind to negatively charged bacterial surfaces. This could be a result of electrostatic binding between the cationic AgNPs and the polyanionic glycopolymers known as WTAs on the surfaces of Gram-positive bacteria [18]. These features define the capability of AgNPs to interact with the negatively charged lipids of bacterial membranes [50], leading to permeabilization and membrane outburst of cell contents. Therefore, we hypothesize that binding between AgNP cations and negatively charged cell wall polymers is determined by electrostatic interactions and that WTAs and related CWGs (which contain negatively charged backbones) [21] might play a major role in disrupting the equilibrium of cell wall dynamics [51]. However, the elaborate processes that interrelate these mechanisms are not fully understood. Future research should focus on defining the molecular details of the key mechanisms and their importance to the antimicrobial activity of AgNPs.

We also propose that Ag^0^/Ag^+^ makes AgNP solutions even more effective in inactivating bacteria. The positively charged AgNPs not only possess a greater affinity to bind to the negatively charged bacterial wall, but these charged particles are also outstanding in Ag^+^ leaching. Therefore, the combination of all three species is more efficient at binding and lysing bacteria. A third species in the AgNP solution is represented by AgNPs that are surrounded by silver ions [52] (adsorbed silver ions on the AgNPs, Ag^0^/Ag^+^). Moreover, further research in this area, in particular with regard to bacterial resistance mechanisms against AgNPs, is warranted.

## Conclusion

The average AgNP size was approximately 1 nm. The adsorption of silver ions on AgNPs creates a third species in AgNP solutions. Zeta potential measurements suggest that the positively charged particles are in fact silver nanoparticles with adsorbed ions (Figure 2). This type of nanoparticle makes AgNP solutions effective bactericidal agents due to the greater affinity of these positively charged AgNPs for the cell walls of MSSA and MRSA.

Electron microscopy images show that both MSSA and MRSA strains treated with AgNPs yield electron-dense particles around the negative backbones of their cell walls, specifically on WTAs and related CWGs, where the interactions first occur. AgNPs can lyse both of the bacterial strains by interacting with their cell walls, resulting in imbalances and increased porosity of their cell membranes and a resultant loss of cytoplasmic content. EDS analyses showed the presence of smaller AgNPs in the cytoplasms of cells that possibly interacted with DNA material (Figure 10). Furthermore, the MIC result of 12.5 ppm correlates with our observations of the ultrastructural changes in bacteria that yield the bactericidal effects of AgNPs against MRSA and MSSA.

The possible antibacterial mechanisms of AgNPs against MSSA and MRSA include the following:

1. We hypothesize that the binding of AgNP cations to negatively charged CWGs is determined by electrostatic interactions between WTAs and related polyanionic CWGs [21] and that these interactions play a key role in creating instability in cell wall physiology [51]. This is because WTAs are located outside of the PG layer [18,20] and are attracted to metal cations [22] with high affinity [20,53].

2. We also propose that the presence of Ag^0^/Ag^+^ makes AgNP solutions even more effective in inactivating bacteria. Positively charged AgNPs not only possess a greater affinity in binding to the negatively charged bacterial wall but also excel in Ag^+^ ion leaching. Therefore, the combination of all three species is more efficient in binding and lysing bacteria.

## Experimental

**Chemicals and Materials:** Silver nitrate, AgNO_3_ (99.99%), was purchased from Sigma-Aldrich and used as received. Distilled water was purified using Whatman^®^ 0.2 µm filters. A Milestone Ethos EZ Microwave Digestion System was used to perform AgNP synthesis.

**Preparation of AgNPs:** To produce AgNPs, 1.7 g AgNO_3_ was added to 20 mL distilled H_2_O and placed in the microwave. The power was set to 1000 W, and the solution was irradiated for 15 s. The resultant solution was clear and light yellow in color, indicating the formation of AgNPs. The AgNP solution generated 23,000 ppm of silver. The solution was kept at room temperature in a dark container to avoid particle aggregation. A drop (≈10 µL) of the resultant solution was placed on a Cu grid (300 mesh) containing a thin carbon film for further analysis. The use of microwaves to synthesize silver nanoparticles has been shown to work in the presence of an eco-friendly reducing agent [54]. Silver nitrate can decompose into metallic silver, NO_2_ gas and O_2_ by the addition of heat as represented in Equation 1 [55]:

[1]
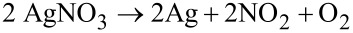


In our study, microwave irradiation generated thermal energy that was able to convert silver nitrate into metallic silver. In such a manner, we are able to produce silver nanoparticles in water without the introduction of contaminants and toxic chemicals, such as borohydride or chloride.

**Determination of particle size:** The size and distribution of AgNPs were assessed by TEM (JEM2100; JEOL, Japan) and ImageJ software by manually measuring the size of separate AgNP particles individually from the TEM images of well-dispersed particles on the sample mount. The average particle size was calculated by a log–normal fit to the size distribution histogram using Origin 2015.

**Culture preparation:** The bacterial strains MSSA (UAMS1) and MRSA (TCH1516) were cultured at 37 °C for 24 h on selective plates (ChromAgar BD Biosciences). Stock cultures were stored at −80 °C in Brain Heart Infusion Agar or BHI (Difco) with 50% glycerol.

**Determination of minimal inhibitory concentration (MIC):** To assess the growth inhibition of the bacterial strains MSSA (UAMS1) and MRSA (TCH1516), they were submitted to determination of the minimal inhibitory concentrations (MICs) of AgNPs by serial dilution in 96-well plates in a similar manner as described in [56] and as suggested by the CLSI (2011). In brief, single colonies of a microbiological culture were grown in selective chromogenic medium plates (ChromAgar BD Biosciences). The colonies were diluted in saline solution and adjusted to 1.5 × 10^8^ CFU/mL. The bacterial suspensions were diluted in Mueller–Hinton broth (Difco, Detroit, Mich.) and plated in flat-bottom polystyrene 96-well tissue culture plates at a final density of 5.0 × 10^5^ CFU/well. Two-fold serial dilutions of AgNPs were prepared at a final volume of 100 µL per well. The final concentration of the AgNPs ranged from 0.4 to 23 ppm; medium without AgNPs served as the nontreated control, and medium alone served as the blank control. The plates were incubated at 37 °C for 18 h, and the optical density values at 600 nm were determined using a microplate reader. All assays were carried out in duplicate, and the experiments were repeated at least three times.

**Sample preparation for STEM:** After 24 h at 37 °C and after adding the bacterial culture (1.5 × 10^8^ CFU/mL) and mixing it with the AgNPs, the samples were centrifuged for 10 min at 3500 rpm. The resultant bacterial pellets were each resuspended in 5 mL PBS and spun down again for 10 min for washing. After washing two times, fixation of the bacterial cells was performed by resuspending each pellet in 1 mL of 4% formaldehyde and 1% glutaraldehyde in PBS. After 2 h of incubation at room temperature, the samples were stored at 4 °C until they were stained with 1% osmium tetroxide (OsO_4_) at room temperature. After washing the bacterial cells with PBS to eliminate excess OsO_4_, a dehydration series was performed with 25, 50, 75, 95 and 100% ethanol. The samples were further dehydrated with propylene oxide, embedded in a resin (LX112) and left to harden for 48 h at 60 °C. The resin capsules were cut using an ultra microtome (Leica Ultracut, UCT) and a 45° diamond knife. Ultrathin sections of approximately 95 nm were obtained and observed using STEM mode.

**Electron microscopy characterization:** Silver nanoparticles were characterized using a 2010-F JEOL field emission transmission electron microscope operated at 200 kV. HAADF-STEM bacterial cell images were obtained using Cs-corrected JEOL JEM-ARM-200F microscope in both bright-field (BF) and dark-field (DF) modes. The microscope was operated at 200 kV using a convergence angle of 26 mrad and collection semi-angles between 50 to 180 mrad. The probe size used was approximately 0.09 nm, and the probe current was 22 pA. To identify the presence of silver in the treated samples, energy dispersive X-ray spectroscopy was performed using a solid state EDAX EDS detector. A Hitachi 5500 SEM was used at 30 kV to collect SE images.
